# Are some interoceptive sensibility components more central than others? Using item pool visualisation to understand the psychometric representation of interoception

**DOI:** 10.1371/journal.pone.0277894

**Published:** 2022-12-01

**Authors:** Jennifer Todd, Viren Swami, Jane E. Aspell, Adrian Furnham, George Horne, Stefan Stieger

**Affiliations:** 1 School of Psychology and Sport Science, Anglia Ruskin University, Cambridge, United Kingdom; 2 Centre for Psychological Medicine, Perdana University, Serdang, Malaysia; 3 Department of Leadership and Organizational Behaviour, Norwegian Business School, Oslo, Norway; 4 Department of Psychology, University of Bath, Bath, United Kingdom; 5 Department of Psychology and Psychodynamics, Karl Landsteiner University of Health Sciences, Krems an der Donau, Austria; Shandong University of Science and Technology, CHINA

## Abstract

Interoception refers to the processing of stimuli originating within the body and is widely considered a multidimensional construct. However, there remains a lack of consensus regarding the definition and measurement of the subjective, self-reported component, referred to here as interoceptive sensibility. As a contribution to knowledge on the topic, we sought to examine the construct commonality and distinguishability of seven self-report measures of interoceptive sensibility using Item Pool Visualisation (IPV), an illustrative method that locates item pools from within the same dataset and illustrates these in the form of nested radar charts. Adults from the United Kingdom (*N* = 802) completed seven measures of interoceptive sensibility, and the data were subjected to IPV. Results demonstrated that, of the included measures, the Multidimensional Assessment of Interoceptive Awareness-2 provided the closest and most precise measurement of the core interoceptive sensibility construct (i.e., core of the entire investigated item pool). The Body Awareness Questionnaire and the Private Body Consciousness Scale were also centrally located measures, while the Body Perception Questionnaire and the Body Responsiveness Scale appear to tap more distal aspects of the core construct. We discuss implications for interpreting complicated data patterns using measures of interoceptive sensibility and, more generally, for measuring the construct of interoceptive sensibility.

## 1. Introduction

*Interoception* refers to a collection of processes through which the internal physiological state of the body is represented by the brain [1]. These processes include the sensing, interpretation, integration, and regulation of internal bodily signals [2]. Interoception has been acknowledged as an important component of different mental health conditions, including anxiety disorders, eating disorders, addictive disorders, and somatic symptom disorders [1].

When operationalising interoception for research, scholars have proposed various components of interoception that appear to represent distinct processes or abilities. Notably, Garfinkel and colleagues [3] proposed the tripartite measurement model of interoception, which comprises *interoceptive accuracy* (i.e., the ability to accurately detect internal bodily sensations; assessed via behavioural tasks), *interoceptive sensibility* (i.e., the self-evaluated assessment of subjective interoception; assessed via self-report measures), and *interoceptive awareness* (i.e., the metacognitive correspondence between objective interoceptive accuracy and self-reported confidence in one’s behavioural performance).

Subsequently, interoception has been widely acknowledged to be a multidimensional process (e.g., [4, 5]) and several variations on the tripartite model have been proposed. Across these models, the subjective sensibility component has been one of the most difficult to define. For example, in the 8-component model proposed by Khalsa and colleagues [1], a slight distinction was made between interoceptive sensibility (defined as the ability to focus on interoceptive stimuli) and *interoceptive self-report scales*, which they defined as the “ability to reflect upon one’s autobiographical experiences of interoceptive states, make judgments about their outcomes, and describe them” (p. S6). Conversely, in their 2 x 2 factorial model of interoception, Murphy and colleagues [6, 7] only distinguished between interoceptive accuracy and interoceptive *attention*, which refers to the degree to which interoceptive signals are the object of attention. Murphy and colleagues [6, 7] further suggested that self-report questionnaire measures could be used to quantify both interoceptive attention (e.g., via the Body Perception Questionnaire; BPQ [8]) and interoceptive accuracy (e.g., via the Interoceptive Accuracy Scale [7]). Finally, Suksasilp and Garfinkel [9] proposed an alternative 8-component theoretical framework for assessing general functioning and individual differences in interoception, which included three components that could be examined via self-report measures: *self-report and interoceptive beliefs* (which broadly encapsulate beliefs concerning interoceptive sensations and experiences, including those derived from self-report measures, such as questionnaires and confidence ratings, and task-based measures of prior beliefs thought to influence perception); *interoceptive attention* (which matches the Murphy et al. definition [7], except for the addition that this component refers to both purposeful attention and the habitual tendency to attend to interoceptive sensations), and; *attribution of interoceptive sensations* (which refers to the interpretation of interoceptive sensations and their causes, such as perceived threat).

Some of the difficulties in defining the subjective component of interoception (referred to from this point on as *interoceptive sensibility*) may stem from attempts to encompass the vast range of measures that purportedly all tap interoceptive sensibility. Indeed, in a recent review, Desmedt and colleagues [10] identified 14 survey-based instruments that have been used to operationalise interoceptive sensibility, although they did not include several questionnaire measures with items that might tap the same construct or, at least related constructs, such as the Body Responsiveness Scale [11], the Interoception Sensory Questionnaire [12], or the Interoceptive Confusion Questionnaire [13]. While the understanding of the associations between these measures remains relatively nascent [10], one theoretical possibility is that interoceptive sensibility itself is multidimensional, and may be best represented by several different constructs (e.g., the measurement models proposed by Suksasilp & Garfinkel [9] or Murphy et al. [6, 7]). For example, unique associations with outcome variables have been identified across different measures of interoceptive sensibility, such as the Multidimensional Assessment of Interoceptive Awareness questionnaire (MAIA [14, 15]) versus the BPQ (e.g., [16–18]). These results would be difficult to synthesise if interoceptive sensibility is considered to be unidimensional. Indeed, historically, different questionnaire measures have been used interchangeably, providing conflicting patterns of results under the unidimensional conceptualisation (e.g., [18]; or [19] *vs*. [20]).

It is also possible that interoceptive sensibility might be better represented as a multilevel construct, with many discernible lower-order constructs that can be examined by different questionnaire measures or different pools of items across existing questionnaire measures. In this conceptualisation, the lower-order facets may represent slightly distinct aspects of the construct (e.g., a distinction between attention and attribution, as in [9]) but are, nonetheless, still considered to be part of a global interoceptive sensibility construct (i.e., subjective assessments of one’s perception of internal bodily processes). As one example, the MAIA (and the revised MAIA-2) purportedly comprise eight different aspects of interoceptive sensibility in the original English language format (namely: noticing, not distracting, not worrying, attention regulation, emotional awareness, self-regulation, body listening, and trusting), which have been differentially associated with several outcome variables (e.g., [16, 21–25]; for a review, see [26]). Nevertheless, Ferentzi and colleagues [27] also found acceptable model fits for the MAIA using both a higher-order and a bifactor model, suggesting that the items and subscales, respectively, also load onto a general or global interoceptive sensibility factor.

To address some of the questions relating to construct dimensionality, Desmedt and colleagues [10] recently examined associations between five commonly used measures of interoceptive sensibility: the BPQ [8], the MAIA-2 [15], the Body Awareness Questionnaire (BAQ [28]), the Private subscale of the Body Consciousness Questionnaire (PBCS [29]), and the Self-Awareness Questionnaire (SAQ [30]). Overall, correlations between the measures ranged in magnitude from *r* = .03 (SAQ–MAIA-2 total score) to *r* = .55 (BAQ–MAIA-2 total score), suggesting that some measures were very distinct from one another, while others may be assessing related yet sufficiently distinct constructs to warrant separate measurement. The authors also explored whether the measures tapped a unified latent construct using exploratory factor analyses. Here, the authors found that the items from the five measures loaded onto five factors. Each of the five factors largely comprised items from one of the five measures under examination, and were named as follows: Neutral and Negative Body Sensations awareness (which included all 26 BPQ Body Awareness scale items), Adaptive Interoception (which included 25 of the 37 MAIA-2 items, collapsing seven of the eight MAIA-2 scales–Not Distracting items were excluded), Negative Feelings Propensity (which included 34 of the 35 SAQ items), Extero-interoceptive Awareness (which included two of the 5 PBCS items pertaining to hunger and temperature sensations, and 17 of the 18 BAQ items), and Interoceptive Not-Distracting (which included three of the six items from the MAIA-2 Not Distracting scale). However, a multilevel (higher-order or bifactor) approach was not included in these analyses, so it is not clear if these factors could be conceptualised as falling under one higher-order or global interoceptive sensibility factor. Finally, the authors explored whether the questionnaire items could be grouped as a unitary network or whether they fell into distinct clusters using network analysis. Seven relatively distinct communities (“subnetworks”) of item nodes were detected in the analysis, suggesting that the communities of items were tapping relatively distinct aspects of the interoceptive sensibility construct.

Overall, the work of Desmedt and colleagues [10] provides initial evidence suggesting that interoceptive sensibility is a multidimensional construct, with several relatively distinct aspects. Important questions following on from this work are: can these aspects of interoceptive sensibility be pragmatically grouped under a global interoceptive sensibility construct, and further, are some aspects more central to this construct than others? The implication of this multilevel conceptualisation is that one could use a ‘general’ indicator of interoceptive sensibility (i.e., a measure that broadly captures subjective assessments of one’s perception of internal bodily processes in a generalised way), or select a measure that targets a more specific aspect of the construct (e.g., the type of attention–maladaptive or adaptive–directed toward interoceptive signals). Put differently, given the plethora of measures available to examine the interoceptive sensibility construct, how do researchers decide which one(s) should be utilised in future research? Primary considerations here might relate to the psychometric properties of the measures (i.e., the validity and reliability of instrument scores in the target population) and practical considerations, such as minimising participant fatigue and research costs. However, when selecting measures for inclusion in a study, it is important to be sure that the measures are not redundant because they measure the same latent factor and that they are sufficiently representative of the aspect of interoceptive sensibility that the researcher wants to investigate.

Here, we suggest that using Item Pool Visualisation (IPV [31]) could be a useful approach for investigating construct commonality and distinguishability, by illustrating comparisons of facets both within and between measures that aim to assess interoceptive sensibility. IPV is a visual tool that uses different structural equation modelling (SEM) estimations to locate items and item pools from within the same dataset. In IPV, models are compared under two conditions: a single factor SEM, where all items are grouped under a global factor that represents the core of the item pool, and; a correlated-factor SEM, where items are grouped under their original measures, and the measures are all inter-correlated without a global factor. Factor loadings from the two models are used to compute *centre distances*, which summarise the ratio of squared item loadings from the two models (i.e., the squared loadings of the correlated factor model are divided by the squared loadings of the general factor model, minus 1 for easier interpretation). Accordingly, centre distances comprise both core variance and additional variance, and represent the uniqueness of each item or group of items relative to the shared core variance of the total item pool. A centre distance close to zero suggests that an item (or group of items) is a strong indicator of the core concept. Conversely, a larger centre distance suggests that an item (or group of items) measures a facet of the construct that is more distinct from the core concept that the whole group of measures purportedly assess. To summarise, in IPV, SEMs are compared to identify the degree to which items or measures are representative of the entire investigated item pool. It is important to note that the core, therefore, does not necessarily represent the core concept in theory–it can only represent the core of the item pool that is being investigated.

In IPV, information is visualised in the form of radar charts. The radar not only illustrates comparisons of scales (such as in correlated factor models), it can also illustrate superordinate commonalities (such as visualisations of general or hierarchical factor models). That is, IPV uses a nested model system, which compares factor loadings when an item pool is divided into sub-pools of items. This method facilitates item pool comparisons across multiple hierarchical levels: sub-pools (facets) can contain further sub-pools, and item pools (questionnaires) can also be combined with other item pools (questionnaires) to form a superordinate item pool. The combination of item- and scale-specific information in radar charts could facilitate the discovery of additional similarities and differences between measures of interoceptive sensibility that may have been overlooked in previous scale comparisons (e.g., [10]).

### 1.1. The present study

The aim of the present study was to contribute to existing knowledge of interoceptive sensibility by examining the item and scale commonality and distinguishability of seven self-report measures using IPV. Item-based analyses were used to examine the degree to which each item represented the core construct of interoceptive sensibility and to examine whether there were problems with some items (e.g., items that might be distant from the rest of the items in each scale). Scale-based analyses were used to determine which measure was closest to the core concept of interoceptive sensibility; that is, to identify if there is a measure that is a good representation of the central construct, which could be selected if a researcher has limited space in a research study. Scale-based analyses were also used to identify measures that are more distant from the core construct (i.e., measures that tap a more unique facet of interoceptive sensibility).

We aimed to include a group of measures that could be considered as indices of distinct yet related aspects of interoceptive sensibility, and that would provide broad coverage of the construct. To that end, we included the four most widely cited measures of interoceptive sensibility (which accounted for a combined 83% of the total citations in the review by Desmedt and colleagues [10]): the Body Awareness subscale from the BPQ [8], the MAIA-2 [15], the PBCS [29], and the BAQ [28]. The BPQ, PBCS, BAQ and MAIA-2 Noticing subscale all have items that measure the tendency to be aware of internal bodily sensations. We hypothesised that the PBCS (comprised of five items that all tap the tendency to notice common internal bodily sensations) would be closest to the core of this item pool (i.e., the concept of interoceptive sensibility, as defined by this set of measures). This was based on the clarity of the items and the consistent pattern of correlations with other measures in previous research [10]. We also expected moderate intercorrelations between the MAIA-2 Noticing scale, the BAQ, and the BPQ (after [10]), and that these measures would also be relatively adept at measuring the central concept, though perhaps the BPQ more distally. This expectation was again based on evidence from previous correlations [10] and on the items in the scale (many of which tap the noticing of uncomfortable body sensations and less common body sensations, such as those associated with illness).

We also included some additional questionnaire measures, which we expected to be more distal measures of the core construct, compared to the measures outlined above. Specifically, the Body Responsiveness Scale (BRS [11]), and the MAIA-2 Attention Regulation, Self-Regulation, Body Listening, and Trusting subscales all tap mindful modes of attention and responses to internal bodily signals, which is slightly distinct from the central concept of noticing internal bodily signals. In previous research, the BRS and BAQ were moderately correlated [11], suggesting slight construct overlap, though the measures are nonetheless nomologically distinct. Equally, the MAIA-2 Not-Worrying and Not-Distracting subscales–which measure maladaptive attention and responses to negative body sensations–have been differentiated from both the other scales of the MAIA/MAIA-2 and other interoceptive sensibility measures (e.g., [10, 27, 32, 33]; see also [34] for a review). Accordingly, we expected these scales to be moderately distal from the core construct.

Finally, we included two measures that tap (to a moderate or strong degree) self-perceived interoceptive accuracy to determine whether these questionnaire measures can be considered part of a broad interoceptive sensibility construct (which include general questionnaire measures of interoception, as in the frameworks outlined in [1, 3]), or whether the self-reported accuracy component of interoception is distinct (see the frameworks outlined in [6, 7, 9]). First, we included the Interoceptive Accuracy Scale (IAS [7]), which measures self-perceived acuity of interoceptive perception. According to Murphy and colleagues’ conceptualisation of interoception [6], self-reported acuity of interoceptive perception is distinct from beliefs regarding the tendency to pay attention to interoceptive signals, and this was demonstrated via a lack of significant correlation with BPQ scores ([7], see also [35]). Consequently, we expected that the IAS would be distal from the core construct. Second, we included the Interoception Sensory Questionnaire (ISQ [12]), which measures the level of difficulty or confusion when sensing interoceptive signals, based upon the specific challenges experienced by people with autism. It includes items that tap the inability to accurately perceive interoceptive signals and some items that tap the tendency not to notice interoceptive signals. Given the focus on self-reported (in)accuracy and the fact that the measure was designed for to tap the difficulties experienced by a specific subpopulation, we also expected this measure to be distally related to the core construct.

## 2. Method

### 2.1. Participants

All participants (*N* = 802) were citizens and residents of the United Kingdom who responded to an online advertisement. The sample was balanced in terms of gender identity (50% women), ranged in age from 18 to 92 years old (*M* = 36.58 ± 12.41), and ranged in self-reported body mass index (BMI) from 14.90 to 49.94 kg/m^2^ (*M* = 28.25 kg/m^2^ ± 7.40). The majority of participants self-reported their ethnicity as British White (88.4%; British Black or African Caribbean = 2.7%; British Asian = 5.5%; mixed race = 2.5%; other = 0.9%). Finally, 13.7% had obtained their General Certificates of Secondary Education (GCSEs), 23.4% had completed an Advanced-Level (A-Level) qualification, 41.9% had an undergraduate degree, 18.3% had a postgraduate degree, 0.2% were in full-time higher education, and 2.4% had some other qualification.

### 2.2. Measures

#### 2.2.1. The short-form body perception questionnaire (bpq [8])

The 26-item Body Awareness Scale from the Short-Form BPQ examines the level of awareness of bodily sensations, including negative body sensations, and sensations associated with illness (sample item: “During most situations I am aware of stomach and gut pains”). Items were rated on a 5-point scale, ranging from 1 (*never*) to 5 (*always*), and mean scores from total item responses were computed so that higher scores reflect greater body awareness. Cabrera and colleagues [36] reported that the short-form Body Awareness Scale had a unidimensional factor structure, good levels of composite reliability (Cabrera et al reported that categorical omega ranged from .83 to .91) and test-retest reliability (ICC = .99), and good patterns of convergent validity.

#### 2.2.2. The Multidimensional Assessment of Interoceptive Awareness-2 (MAIA-2 [15])

The 37-item MAIA-2 is a revision of the MAIA [14], with five new items added across the Not-Worrying and Not-Distracting subscales. The Noticing subscale assesses the subjective awareness of body sensations (4 items, sample item: “When I am tense, I notice where the tension is located in my body”). The Not-Distracting subscale assesses how often a person tends to ignore sensations of pain or discomfort (6 items, sample item: “I ignore physical tension or discomfort until they become more severe”). The Not-Worrying subscale assesses the extent to which a person worries about or catastrophises sensations of pain or discomfort (5 items, sample item: “When I feel physical pain, I become upset”). The Attention Regulation subscale assesses the ability to control and maintain attention towards bodily sensations (7 items, sample item: “I can pay attention to my breath without being distracted by things happening around me”). The Emotional Awareness subscale assesses the awareness of the relationship between emotional and bodily states (5 items, sample item: “I notice how my body changes when I am angry”). The Self-Regulation subscale assesses whether a person uses attention to bodily sensations to regulate distress (4 items, sample item “When I feel overwhelmed, I can find a calm place inside”). The Body Listening subscale assesses how often a person actively attends to their bodily sensations for insight (3 items, sample item “I listen for information from my body about my emotional state”). Finally, the Trusting subscale assesses the extent to which a person experiences their body as a ‘safe’ and ‘trustworthy’ source of information (3 items, “I trust my body sensations”). Items were rated on a 6-point scale, ranging from 0 (*never*) to 5 (*always*), and mean scores were computed for each subscale so that higher scores indicate greater interoceptive sensibility. Mehling and colleagues [15] reported that the MAIA-2 has an 8-factor structure (although several model fit indices were less than ideal),.Six subscales had adequate levels of composite reliability (Mehling et al reported that Cronbach’s α ranged from .74 to .83), and two subscales had less than ideal levels of composite reliability (Mehling et al reported that Cronbach’s α = .64, and .67 for the Noticing and Not-Worrying scales, respectively [15]). The MAIA and MAIA-2 have good patterns of convergent and discriminant validity [14, 15], and can distinguish between groups of adults with training in mindfulness techniques [14, 26, 37].

#### 2.2.3. The Private Bodily Consciousness Scale (PBCS [29])

The 5-item PBCS from Body Consciousness Questionnaire [29] examines the level of sensitivity to bodily sensations including thirst, hunger, heartbeats and temperature (sample item: “I’m very aware of changes in my body temperature”). Items were rated for the extent to which they are characteristic of the respondent on a 5-point scale, ranging from 0 (*extremely uncharacteristic*) to 4 (*extremely characteristic*), and overall scores were computed as the mean of all items so that higher scores indicate greater interoceptive sensibility. The PBCS has good patterns of reliability and validity in clinical samples [38], and acceptable composite reliability and test-rest reliability (α < .80, test-retest *r* = .69 in previous samples) [29, 38].

#### 2.2.4. The Body Awareness Questionnaire (BAQ [28])

The 18-item BAQ was designed to measure self-reported attentiveness to bodily processes, such as body cycles and rhythms, attentiveness to changes in normal functioning, and the ability to anticipate bodily reactions (sample item: “I notice differences in the way my body reacts to certain foods”). Items were rated on a 7-point scale, ranging from 1 (*not at all true about me*) to 7 (*very true about me*). Overall scores were computed as the mean of all items, where higher scores indicate greater body awareness. Shields and colleagues [28] reported that factor analysis indicated the BAQ has four sub-domains, which are not scored separately: Note response or changes in body process (sample item: “I notice differences in the way my body reacts to various foods”), Predict body reactions (sample item: “Whenever my exercise habits change, I can predict very accurately how that will affect my energy level”), Sleep-wake cycle (sample item: “There seems to be a ‘best’ time for me to go to sleep at night”), and Prediction of the onset of illness (sample item: “I know I’m running a fever without taking my temperature”). Shields and colleagues [28] also reported that the BAQ has appropriate composite reliability (Shiels et al reported that α ranged from .77 to .83) and test-rest reliability over a 2-week period (*r* = .80), and that the BAQ has good patterns of convergent and discriminant validity. Multiple additional studies have supported reliability, convergent, and discriminant validity (for an overview, see [38]).

#### 2.2.5. The Body Responsiveness Scale (BRS [11])

The 7-item BRS is a measure of the level of attunement that an individual has to their body’s needs and the extent to which they use embodied information to guide behaviour (sample item: “I enjoy becoming aware of how my body feels”). Items were rated on a 7-point scale ranging from 1 (*not at all true about me)* to 7 (*very true about me*). An overall score was computed as the mean of all 7 items following reverse coding of 3 items, so that higher scores reflect greater body responsiveness. Daubenmier [11] reported that BRS scores had adequate composite reliability (α = .83), and adequate patterns of construct validity in English-speaking adults.

#### 2.2.6. The Interoception Sensory Questionnaire (ISQ [12])

The 20-item ISQ was designed to capture variance in interoception difficulties that are specific to the experience of people with autism. The ISQ measures confusion about interoceptive bodily states unless these states are extreme (sample item: “I have difficulty making sense of my body’s signals unless they are very strong”). Items were rated on a 7-point Likert scale, ranging from 1 (*not at all true of me*) to 7 (*very true of me*), and mean scores were computed so that higher scores represent increased interoceptive challenges. Fiene and colleagues [12] reported that the ISQ has a unidimensional factor structure and adequate composite reliability (α = .96), and preliminary evidence showed good patterns of convergent and discriminant validity. The measure was also able to distinguish between groups of adults with and without a diagnosis of autism [12].

#### 2.2.7. The Interoceptive Accuracy Scale (IAS [7])

The 21-item IAS examines the self-reported accuracy with which bodily sensations are perceived (sample item: “I can always accurately perceive when my heart is beating fast”). Items were responded to on a 5-point scale, ranging from 1 (*disagree strongly*) to 5 (*strongly agree*). Overall scores were computed as the mean of all item responses, where higher scores correspond to greater self-reported interoceptive accuracy. Using principal components analysis and confirmatory factor analysis, Murphy and colleagues [7] tested 1-factor and 2-factor models. They found that the 2-factor model was slightly preferential, though model fit indices for both models were less than ideal. The authors found that IAS total scores had adequate composite reliability (α = .88 - .90) and test-retest reliability over a 30-day period (*r* = .75), and good patterns of convergent and discriminant validity [7].

#### 2.2.8. Demographic items

Participants were asked to self-report their gender identity, age, ethnicity, and highest educational qualifications. We also asked participants to self-report their height and weight, which we used to compute BMI as kg/m^2^. These data were used for sample-descriptive purposes only.

### 2.3. Procedure

Prior to conducting the research, ethics approval was obtained from the relevant school research ethics committee at Anglia Ruskin University (approval code: EHPGR-28). The project was advertised as a study “assessing bodily awareness” and included an estimated duration of 15 minutes. Participants were recruited via the Prolific website, a crowdsourcing internet marketplace that allows individuals to complete surveys for monetary compensation. Inclusion criteria included being a citizen and resident of the United Kingdom, self-reported fluency in English comprehension and ability to complete a survey in English, and being of adult age. These criteria were included to ensure that the sample is homogeneous in terms of cultural and national identity, both of which could have an impact on interoceptive sensibility [34, 39]. IP addresses were checked to ensure that no participant completed the survey more than once and data were screened to ensure that no participants failed the following attention check item, which was embedded in the survey as a multiple-choice question: “The animal test you are about to take part in is very simple: when asked your favourite animal, you must select ‘frog’. This is an attention check. Based on the text you read above, what animal have you been asked to select?” Participants were paid £1.25 for their time, which is commensurate with Prolific payment recommendations based on survey length. Once participants provided digital informed consent, they were asked to complete an anonymous survey containing the measures described above, which were presented in a counter-balanced order for each participant. Next, participants provided their demographic information before receiving written debriefing information.

### 2.4. Statistical analyses

#### 2.4.1. Preliminary analyses

Improbable BMI values (< 12 or > 50 kg/m^2^; *n* = 62) were removed and treated as missing data. Overall, missing values accounted for 0.06% of the total dataset and were missing completely at random (MCAR), as determined by Little’s MCAR analysis: χ(1285) = 1,290.66, *p* = .450. Missing data were therefore imputed using the multiple imputation technique using the *mice* package [40] in *R* [41]. We used confirmatory factor analyses (CFA) to examine the fit of the parent dimensional models for scores on each of the interoceptive sensibility measures in our dataset. For the CFAs, we used the *lavaan* package [42] with *R*. Assessment of the data for normality indicated that they were neither univariate nor multivariate normal, so parameter estimates were obtained using the robust maximum likelihood method with the Satorra-Bentler correction [43]. To assess goodness-of-fit, we used the normed model chi-square (χ^2^/df; values <3.0 considered indicative of good fit and values up to 5.0 considered adequate [44]), the Steiger-Lind root mean square error of approximation (RMSEA) and its 90% confidence interval CI (values close to .06 are considered to be indicative of good fit and values of about .07 - .08 indicative of adequate fit [45]), the standardised root mean square residual (SRMR; values < .09 indicative of reasonable fit [44]), and the comparative fit index (CFI; values close to or >.95 indicative of adequate fit [44]). Where models demonstrated less-than-adequate fit, suggested modification indices were considered to improve model fit.

#### 2.4.2. Item pool visualization

IPV analyses were computed using the *IPV* package [46] in *R* [41], and structural equation models (SEMs) were calculated using the *lavaan* package [43]. The planned analytic strategy for the IPV analyses followed the procedural steps outlined by Dantlgraber and colleagues [31] and Swami and colleagues [47]. Briefly, we planned to first produce a general factor model of interoceptive sensibility using SEM (i.e., a single factor extracted from the overall item pool). This factor would be taken to represent the “core concept” of interoceptive sensibility. At first inspection, the ISQ items had very low path coefficients, with most of the loadings < |0.1|, which suggests that the ISQ is very distant from the central construct. Consistent with this, the correlations between the ISQ and the pool of other measures were also low (see Table 2). These findings were in accordance with our hypotheses, but we nevertheless elected to remove the scale from further analyses at this stage to ensure that the central representation of the construct was not skewed.

Next, we computed a new general factor model excluding the ISQ items. Here, and in the previous model outlined above, we found that reverse-coded items in the MAIA-2 (Items #5 through 12 and 15) and the BRS (Items #2, 3, and 4) had substantial negative loadings (the MAIA-2 items ranged from -.130 to -.357, and BRS items ranged from -.107 to -.143), despite recoding the values so that they should load positively. A similar issue was encountered by Swami and colleagues [47] regarding BRS Items #2, 3, and 4, and problems with the MAIA-2 Not Distracting and Not Worrying subscales are well-documented (see [34]). The substantial negative loadings suggest a problem with the items (indeed, BRS items #2, 3, and 4 did not significantly load onto the factor when we attempted to confirm the unidimensional factor structure via CFA; see Table 1), which is probably due to their reversed-coded item formulation. Swami and colleagues [47] suggested that these items should be excluded from the scale if the measure underwent a formal phase of psychometric development. At this stage, therefore, we elected to remove BRS items #2, 3, and 4, and the MAIA-2 Not Distracting and Not Worrying subscales, and proceed with the remaining items.

**Table 1:** Results of confirmatory factor analyses examining fit of the parent models for each of the scales included in the present study.

Scale	SBχ^2^	*df*	χ^2^/*df*	Robust RMSEA (90% CI)	SRMR	Robust CFI
(1) BPQ	1631.74	299	5.26	.084 (.080, .088)	.054	.846
(1a) BPQ with covariances freed for Items 10 and 14	1488.92	298	5.00	.080 (.076, .084)	.052	.862
(1b) BPQ with covariances freed for Items 10 and 14, and Items 13 and 14	1344.05	297	4.53	.075 (.071, .079)	.051	.879
(1c) BPQ with covariances freed for Items 10 and 14, Items 13 and 14 and Items 15 and 16	1277.81	296	4.32	.073 (.069, .077)	.050	.887
(2) MAIA-2	2408.03	601	4.01	.069 (.066, .072)	.086	.859
(2a) MAIA-2 with covariances freed for Items 13 and 14	2030.91	600	3.38	.061 (.059, .064)	.084	.894
(2b) MAIA-2 with covariances freed for Items 13 and 14, and Items 30 and 31	1831.59	599	3.06	.057 (.054, .060)	.082	.903
(3) PBCS*	2.81	5	0.56	.000 (.000, .039)	.012	1.000
(4) BAQ	724.90	135	5.37	.085 (.079, .092)	.062	.807
(4a) BAQ with covariances freed for Items 2 and 3	617.78	134	4.61	.077 (.071, .084)	.057	.843
(5) BRS	446.36	14	31.88	.217 (.200, .235)	.151	.693
(6) ISQ	962.43	170	5.66	.092 (.087, .098)	.056	.857
(6a) ISQ with covariances freed for Items 5 and 13	877.67	169	5.19	.087 (.082, .093)	.055	.873
(6b) ISQ with covariances freed for Items 5 and 13, and Items 19 and 20	814.35	168	4.85	.084 (.078, .090)	.054	.883
(6b) ISQ with covariances freed for Items 5 and 13, Items 19 and 20, and Items 3 and 11	765.30	167	4.58	.081 (.075, .087)	.053	.893
(7) IAS	1099.00	189	5.81	.089 (.084, .094)	.069	.764
(7a) IAS with covariances freed for Items 1 and 3	938.44	188	4.99	.080 (.075, .086)	.064	.808

*Notes*. BPQ = Body Perception Questionnaire, MAIA-2 = Multidimensional Assessment of Interoceptive Awareness-2, PBCS = Private Body Consciousness Scale, BAQ = Body Awareness Questionnaire, BRS = Body Responsiveness Scale, ISQ = Interoception Sensory Questionnaire, IAS = Interoceptive Accuracy Scale. All *p*s < .001, except for the PBCS where *p* = .729.

Next, we estimated correlated factor models based on SEM, where factors were extracted from increasingly restrictive and specific sub-pools of items (i.e., six correlated factors representing the six remaining measures we included in the survey after the removal of the ISQ, where items only loaded onto their respective scale). Given that the MAIA-2 is the only scale with several subscales (all the other measures purportedly have a unidimensional factor structure), we computed separate models using the MAIA-2, where we considered the items and scales in isolation, and a nested model with the MAIA-2 scales considered alongside the other scales. Finally, centre distances were calculated. Centre distances represent the proportional increase of the explained item variance when the items are allocated to smaller sub-pools compared to the larger common pool (see Section 1; see also [31, 47]).

## 3. Results

### 3.1. Preliminary analyses

Results of the CFAs are summarised in Table 1. As can be seen, the factor models for all measures had fits that were less than ideal. For the BPQ, MAIA-2, BAQ, ISQ, and IAS, model fits were improved by including correlated item errors within the models (up to a maximum of three item pairs), which brought fit indices into an acceptable range, although CFI remained below acceptable thresholds for all measures. Fit of the PBCS could not be reliably estimated because the model appeared to be saturated (i.e., the model only seems to fit the data perfectly because it has as many estimated parameters as there are values to be fitted). Although these results suggest that scores for all measures may not match the parent model factor structures in our dataset, the intention of IPV is not to reassess the factorial validity of established measures. As such, we proceeded on the basis of considering each of the aforementioned scales as consistent with the parent model factor structures and used the results of the CFA to explain complications arising in the IPV (see Section 4.). The only exceptions to this were the BRS (we removed Items #2, 3, and 4 before proceeding to IPV) and the MAIA-2 (we removed the Not Distracting and Not Worrying scales before proceeding to IPV), as previously outlined in Section 2.4.

Scores on most instruments demonstrated adequate internal consistency coefficients as indexed using McDonald’s ω (see Table 2). Coefficients were relatively attenuated for BRS scores and PBCS scores, although these findings are consistent with previous work [10, 11]. As can be seen in Table 2, inter-correlations between instrument scores ranged from *r* < .01 (IAS–MAIA-2 Not Worrying) to *r* = .58 (MAIA-2 Noticing–PBCS), but were generally moderate in strength.

**Table 2:** Internal consistency coefficients, descriptive statistics, and inter-correlations between scores on all measures included in the present study.

	McDonald’s ω (95% CI)	*M* (*SD*)	(1)	(2)	(3)	(4)	(5)	(6)	(7)	(8)	(9)	(10)	(11)	(12)	(13)
(1) BPQ	.95 (.95, .96)	2.48 (0.80)													
(2) MAIA-2: Noticing	.76 (.73, .79)	2.80 (0.98)	.42												
(3) MAIA-2: Not-distracting	.89 (.88, .90)	1.98 (1.00)	-.20	-.25											
(4) MAIA-2: Not-worrying	.74 (.71, .77)	2.61 (0.92)	-.16	-.12	-.08										
(5) MAIA-2: Attention regulation	.87 (.86, .89)	2.52 (0.93)	.20	.47	-.27	.13									
(6) MAIA-2: Emotional awareness	.84 (.82, .86)	2.97 (1.11)	.34	.55	-.20	-.15	.46								
(7) MAIA-2: Self-regulation	.85 (.83, .86)	2.42 (1.10)	.19	.36	-.17	.09	.52	.53							
(8) MAIA-2: Body listening	.86 (.84, .87)	1.96 (1.20)	.27	.43	-.11	.09	.46	.56	.61						
(9) MAIA-2: Trusting	.91 (.89, .92)	2.81 (1.25)	.03*	.25	-.05*	.16	.41	.31	.49	.43					
(10) PBCS	.67 (.63, .71)	2.31 (0.69)	.44	.58	-.25	-.16	.33	.41	.20	.29	.13				
(11) BAQ	.87 (.86, .89)	4.17 (0.95)	.26	.48	-.22	-.08	.41	.55	.41	.47	.33	.42			
(12) BRS	.63 (.59, .66)	3.94 (0.93)	.02*	.23	-.04*	.07*	.37	.31	.37	.45	.51	.13	.34		
(13) ISQ	.93 (.93, .94)	2.66 (1.05)	.13	-.08	-.18	-.05*	-.08	-.04*	-.01*	.03*	-.21	-.12	-.11	-.27	
(14) IAS	.88 (.87, .90)	3.83 (0.53)	.06*	.29	-.10	< .01*	.27	.28	.18	.17	.30	.28	.41	.21	-.40

*Note*. CI = Confidence interval, BPQ = Body Perception Questionnaire, MAIA-2 = Multidimensional Assessment of Interoceptive Awareness 2, PBCS = Private Body Consciousness Scale, BAQ = Body Awareness Questionnaire, BRS = Body Responsiveness Scale, ISQ = Interoception Sensory Questionnaire, IAS = Interoceptive Accuracy Scale. All correlations were significant at *p* < .001 except those denoted with * which were not statistically significant at *p* < .05.

### 3.2. Item pool visualisation

#### 3.2.1. Item-based analyses

**Fig 1:**
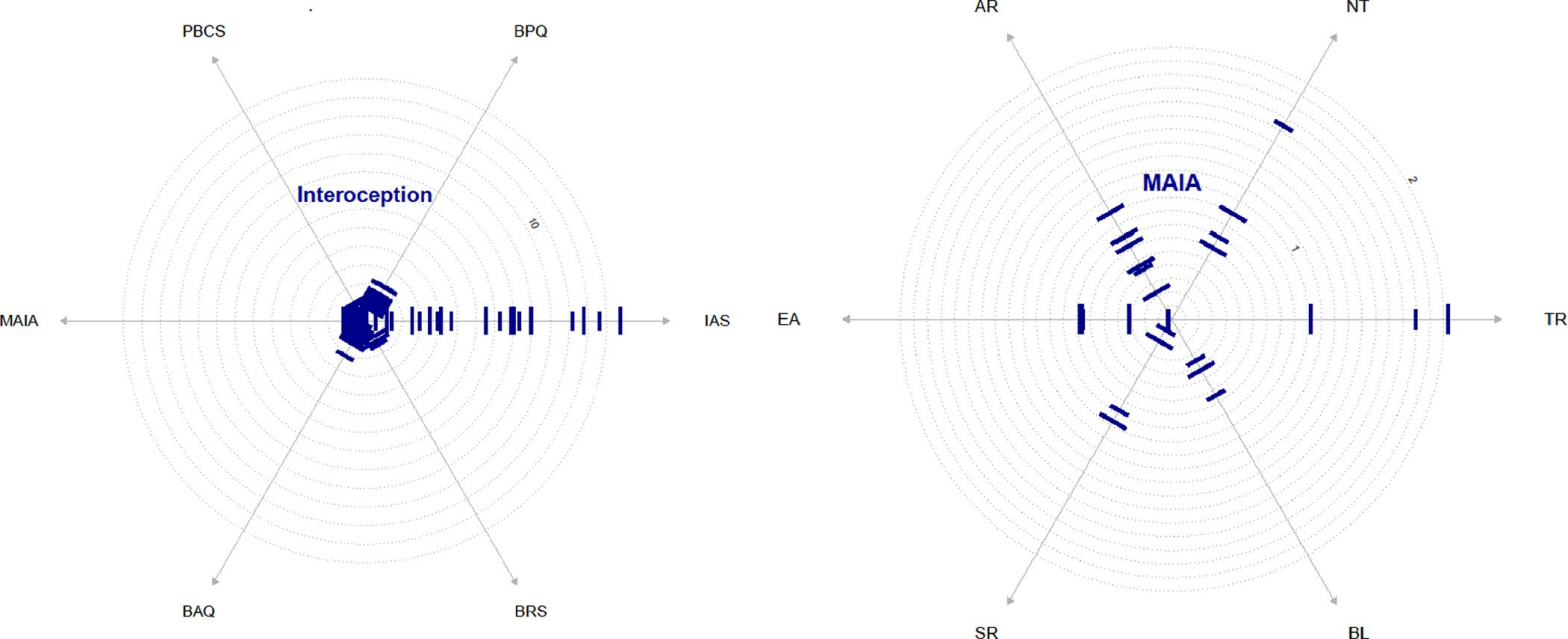
Radar charts with item locations on scale dimensions for all measures (left) and the MAIA-2 subscales in isolation (right). The dotted circles represent the grid of axis scaling. For clearer distinction, every second item is illustrated as having a different length. AR = Attention Regulation, NT = Noticing, TR = Trusting, BL = Body Listening, SR = Self-Regulation, EA = Emotional Awareness.

As outlined above, the outcome from the general factor model represents a “core concept” (i.e., a general factor model) of interoceptive sensibility that all items from all measures are assessing. This is represented by the centre of the radar plots (see Fig 1). Larger centre distances indicate that the item assesses a more facet-specific compared to the general aspects of the core concept that are represented in the centre. Item-based analyses from the whole item pool indicated that the IAS had the most heterogeneous item set compared to the other scales (see Fig 1, left panel and Table 3). While some IAS items were located relatively centrally (e.g., Item #1: “I can always accurately perceive when my heart is beating fast”, centre distance = 0.49), 17 of the 21 IAS items had centre distances > 2.07 (the highest centre distance for an item located in any of the other measures). The item located furthest from the centre was IAS Item #6 (“I can always accurately perceive when I need to defecate”, centre distance = 13.65).

**Table 3:** Basic item pool visualisation calculations for the nested model.

Scale	Item #	Factor loadings		Centre distance	Mean centre distance / Aggregated mean centre distance
		General factor model	Correlated factor model		
BPQ	1	0.340	0.436	0.644	1.165 / 1.140
BPQ	2	0.384	0.591	1.371	
BPQ	3	0.425	0.626	1.171	
BPQ	4	0.409	0.547	0.785	
BPQ	5	0.444	0.710	1.555	
BPQ	6	0.416	0.609	1.143	
BPQ	7	0.492	0.682	0.921	
BPQ	8	0.414	0.721	2.031	
BPQ	9	0.478	0.704	1.166	
BPQ	10	0.491	0.650	0.752	
BPQ	11	0.480	0.601	0.568	
BPQ	12	0.490	0.713	1.117	
BPQ	13	0.520	0.816	1.464	
BPQ	14	0.527	0.758	1.073	
BPQ	15	0.481	0.665	0.909	
BPQ	16	0.433	0.670	1.390	
BPQ	17	0.467	0.720	1.377	
BPQ	18	0.377	0.595	1.488	
BPQ	19	0.428	0.600	0.966	
BPQ	20	0.397	0.547	0.898	
BPQ	21	0.418	0.645	1.381	
BPQ	22	0.475	0.643	0.835	
BPQ	23	0.346	0.598	1.993	
BPQ	24	0.448	0.656	1.137	
BPQ	25	0.491	0.628	0.634	
BPQ	26	0.440	0.699	1.531	
PBCS	1	0.408	0.582	1.032	0.880 / 0.890
PBCS	2	0.412	0.575	0.949	
PBCS	3	0.365	0.463	0.610	
PBCS	4	0.404	0.554	0.881	
PBCS	5	0.368	0.511	0.926	
MAIA-2 –Noticing	1	0.514	0.502	0.000	0.383 / 0.290
MAIA-2	2	0.561	0.458	0.000	
MAIA-2	3	0.535	0.513	0.000	
MAIA-2	4	0.538	0.465	0.000	
MAIA-2 –Attention Regulation	1	0.414	0.501	0.460	
MAIA-2	2	0.515	0.580	0.269	
MAIA-2	3	0.392	0.488	0.544	
MAIA-2	4	0.469	0.582	0.537	
MAIA-2	5	0.531	0.630	0.404	
MAIA-2	6	0.447	0.566	0.602	
MAIA-2	7	0.481	0.611	0.611	
MAIA-2 –Emotional Awareness	1	0.518	0.541	0.090	
MAIA-2	2	0.565	0.531	0.000	
MAIA-2	3	0.577	0.611	0.124	
MAIA-2	4	0.587	0.611	0.084	
MAIA-2	5	0.627	0.664	0.122	
MAIA-2 –Self-Regulation	1	0.435	0.564	0.684	
MAIA-2	2	0.558	0.697	0.557	
MAIA-2	3	0.473	0.619	0.716	
MAIA-2	4	0.509	0.650	0.633	
MAIA-2 –Body Listening	1	0.598	0.692	0.340	
MAIA-2	2	0.571	0.660	0.335	
MAIA-2	3	0.581	0.673	0.339	
MAIA-2 –Trusting	1	0.363	0.523	1.070	
MAIA-2	2	0.391	0.539	0.905	
MAIA-2	3	0.451	0.561	0.544	
BAQ	1	0.506	0.518	0.051	0.809/ 0.667
BAQ	2	0.338	0.427	0.597	
BAQ	3	0.419	0.501	0.433	
BAQ	4	0.508	0.598	0.385	
BAQ	5	0.393	0.542	0.903	
BAQ	6	0.403	0.530	0.728	
BAQ	7	0.393	0.547	0.931	
BAQ	8	0.421	0.626	1.213	
BAQ	9	0.495	0.653	0.741	
BAQ	10	0.018	0.032	2.073	
BAQ	11	0.387	0.559	1.088	
BAQ	12	0.352	0.491	0.943	
BAQ	13	0.498	0.608	0.487	
BAQ	14	0.451	0.507	0.268	
BAQ	15	0.364	0.541	1.210	
BAQ	16	0.485	0.642	0.755	
BAQ	17	0.317	0.483	1.323	
BAQ	18	0.483	0.579	0.438	
BRS	1	0.417	0.613	1.163	1.081 / 1.027
BRS	5	0.462	0.714	1.389	
BRS	6	0.608	0.834	0.885	
BRS	7	0.585	0.804	0.888	
IAS	1	0.346	0.423	0.494	6.065/ 4.303
IAS	2	0.214	0.477	3.993	
IAS	3	0.321	0.492	1.339	
IAS	4	0.228	0.448	2.857	
IAS	5	0.151	0.537	11.691	
IAS	6	0.142	0.542	13.649	
IAS	7	0.246	0.582	4.583	
IAS	8	0.191	0.547	7.179	
IAS	9	0.183	0.555	8.235	
IAS	10	0.195	0.612	8.858	
IAS	11	0.176	0.613	11.103	
IAS	12	0.181	0.540	7.919	
IAS	13	0.196	0.584	7.925	
IAS	14	0.184	0.546	7.771	
IAS	15	0.234	0.637	6.441	
IAS	16	0.278	0.403	1.095	
IAS	17	0.178	0.655	12.564	
IAS	18	0.307	0.307	0.000	
IAS	19	0.297	0.551	2.440	
IAS	20	0.228	0.502	3.831	
IAS	21	0.240	0.503	3.403	

*Note*. BPQ = Body Perception Questionnaire; PBCS = Private Body Consciousness Scale; MAIA-2 = Multidimensional Assessment of Interoceptive Awareness-2; BAQ = Body Awareness Questionnaire; BRS = Body Responsiveness Scale; IAS = Interoceptive Accuracy Scale.

Outside of the IAS items, Fig 1 shows that two other items were distally located relative to the other pools of items, namely, BAQ Item #10 (“I don’t notice seasonal rhythms and cycles in the way my body functions”; centre distance = 2.07) and BPQ Item #8 (“During most situations, I am aware of an urge to defecate”; centre distance = 2.03). This might represent a problem with the validity of these items (e.g., respondents may be less familiar with the term ‘defecate’) or the validity of the scale (indeed, model fit was less-than-ideal for both measures; see Table 2). It is notable that BAQ Item #10 is the only reverse scored item within that scale, which may have some impact on item performance relative to the remaining item pool.

Items located most centrally within the item pool were BAQ Item #1 (“I notice differences in the way my body reacts to various foods”; centre distance = 0.05), and four items from the MAIA-2 Emotional Awareness subscale: Item #4, “I notice that my breathing becomes free and easy when I feel comfortable” (centre distance = 0.08; Item #1, “I notice how my body changes when I am angry” (centre distance = 0.09); Item #5 “I notice how my body changes when I feel happy/joyful” (centre distance = 0.12), and; Item #3 “I notice that my body feels different after a peaceful experience” (centre distance = 0.12). Considered altogether, these items appear to be tapping a “reactive interoceptive sensibility”; that is, they all refer to the tendency to notice bodily changes in response to an event that might change one’s physiological condition (consumption of food, a peaceful experience, a change in emotional state).

Further considering the items from the MAIA-2 in isolation (see Fig 1 right panel, and Table 3), Item #1 from the Emotional Awareness subscale was the most centrally located item (centre distance = 0.02), with Self-Regulation subscale Item #2 (“When I bring awareness to my body I feel a sense of calm”; centre distance = 0.08) and Item #1 (“When I feel overwhelmed I can find a calm place inside”; centre distance = 0.17) also located very centrally. The Trusting subscale items were most distal to the central concept measured by the MAIA-2, with Trusting Item #2 (“I feel my body is a safe place”) having the highest centre distance (2.02). Nevertheless, the MAIA-2 items were generally very uniformly distributed.

It is important to note that caution is required when interpreting the items located most centrally within the total item pool, because all four items from the MAIA-2 Noticing subscale, and IAS Item #18 appear to have centre distances of 0. Centre distances represent the proportional increase of the explained item variance, if the items are allocated to smaller sub-pools compared to a larger common pool. A centre distance of 0 represents no increase, and also includes a possible decrease of explained variance. That is, negative values are treated as 0. Accordingly, the MAIA-2 Noticing Items and IAS Item #8 were set to 0 by the IPV package, because IPV can only be calculated when all centre distances are positive, and these items were negative. In rare cases, some centre distances may switch from a positive to a negative centre distance because they are close to zero, but this is speculative.

#### 3.2.2. Scale-based analyses

**Fig 2:**
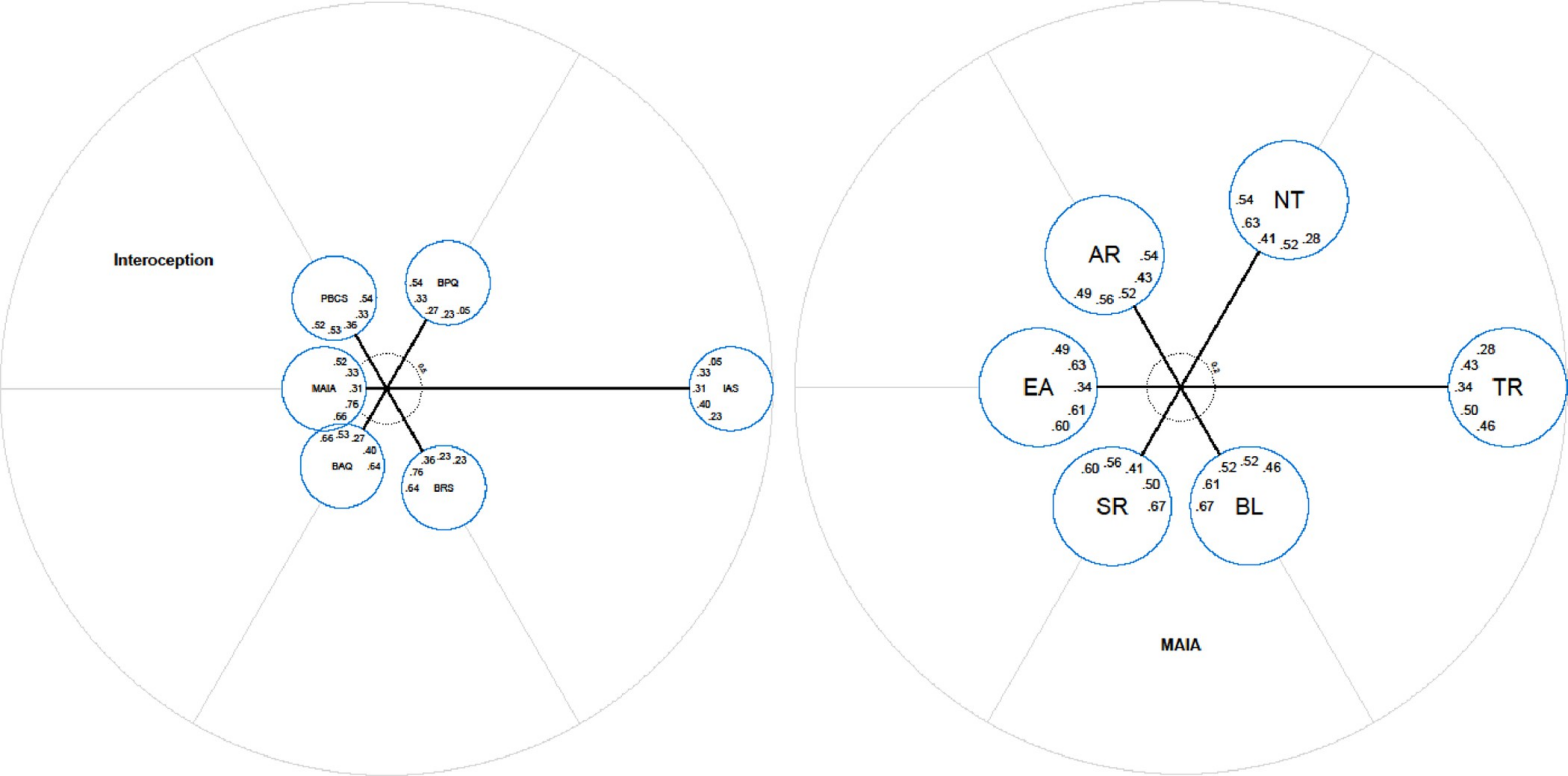
Radar charts with scale locations of all interoceptive sensibility measures (left) including a zoomed chart of the centre, and MAIA-2 subscales in isolation (right). Numbers within the circles represent latent correlations between the respective scale with all the other scales. Correlations are arranged clockwise using the same order as the scales. AR = Attention Regulation, NT = Noticing, TR = Trusting, BL = Body Listening, SR = Self-Regulation, EA = Emotional Awareness.

As can be seen from Tables 3 and 4, in addition to generating centre distances for each item, IPV also generates centre distances for each of the measures (item pools), which are computed as the aggregated centre distance of all items from the respective scale [48]. (In a previous version of IPV, a ‘mean centre distance’ was calculated but this measure was prone to extreme values (i.e., outliers). The newly introduced aggregate centre distance of a specific pool is the relative increase in explained item variance across all items from that pool.) Based on the scale view that illustrates each item pool as a circle (the overall pool and the specific scales; see Fig 2), the MAIA-2 (when considered as a whole) is the most centrally located measure, followed by the BAQ and the PBCS, which are slightly more distal, though nonetheless central measures of the construct. At the next step, the BRS and the BPQ were more distally located. The IAS was furthest from the centre compared to all the other measures. The values within the circles in Fig 2 are the correlations (standardised path coefficients) between the measures, arranged in a clockwise fashion. Again, the MAIA-2 had on average the highest latent correlations with all the other measures, further suggesting that the MAIA-2 may be the best representative of the core construct examined by all of the measures.

**Table 4:** Basic item pool visualisation calculations for multidimensional assessment of interoceptive awareness-2.

MAIA- 2 Subscale	Item #	Factor loadings		Centre distance	Mean centre distance / Aggregated mean centre distance
		General factor model	Correlated factor model		
Noticing	1	0.502	0.688	0.883	0.947 / 0.927
	2	0.458	0.743	1.630	
	3	0.513	0.665	0.682	
	4	0.465	0.586	0.592	
Attention Regulation	1	0.501	0.651	0.690	0.565 / 0.552
	2	0.580	0.698	0.446	
	3	0.488	0.633	0.684	
	4	0.582	0.801	0.894	
	5	0.630	0.800	0.614	
	6	0.566	0.672	0.411	
	7	0.611	0.674	0.216	
Emotional Awareness	1	0.541	0.545	0.016	0.457 / 0.493
	2	0.531	0.606	0.302	
	3	0.611	0.785	0.650	
	4	0.611	0.784	0.647	
	5	0.664	0.858	0.668	
Self-Regulation	1	0.564	0.611	0.173	0.467 / 0.462
	2	0.697	0.724	0.079	
	3	0.619	0.843	0.854	
	4	0.650	0.863	0.762	
Body Listening	1	0.692	0.801	0.339	0.463/ 0.458
	2	0.660	0.843	0.633	
	3	0.673	0.801	0.417	
Trusting	1	0.523	0.872	1.779	1.602 / 1.582
	2	0.539	0.937	2.019	
	3	0.561	0.795	1.008	

*Note*. MAIA-2 = Multidimensional Assessment of Interoceptive Awareness-2

**Fig 3:**
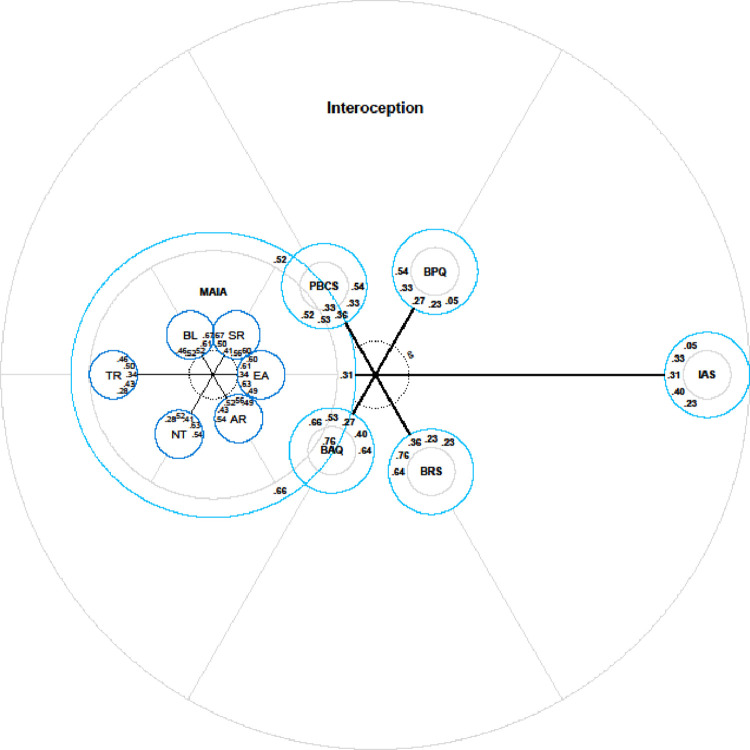
A nested model to demonstrate centre distances within MAIA-2 scales and across all of the measures of interoceptive sensibility. The position of the subscales within MAIA-2 cannot be interpreted relative to the other measures in the chart.

We also generated center distances for the subscales within the MAIA-2. As can be seen from Fig 2 (right panel) and Table 4, the Emotional Awareness, Self-Regulation, Body Listening, and Attention Regulation scales were all located very centrally, with very little difference in center distances. Of this central group, the Emotional Awareness subscale had the smallest center distance. The Noticing subscale was distal to this central group of measures and the Trusting subscale was located furthest from the center compared to all the other subscales. Finally, Fig 3 is a nested model, comprised of the two models from Fig 2 (left and right panels combined). It is important to note that the models represent independent measurement levels; therefore, the position of the MAIA-2 subscales in Fig 3 cannot be interpreted relative to the other measures in the chart (e.g., the BAQ, the BRS, and so on).

## 4. Discussion

Recent research indicates that the self-reported component of interoception–referred to here as interoceptive sensibility–may be a multidimensional construct, with evidence suggesting that some of the most widely cited measures tap related yet relatively distinct facets [10]. In the present study, we examined construct commonality and distinguishability of seven self-report measures of interoception using IPV, with the aim of determining whether these measures might load onto one global interoceptive sensibility factor, and whether some of these measures are more central to the global construct than others.

Overall, we demonstrated that five of the seven measures included in the study (the MAIA-2, the BAQ, the PBCS, the BRS, and the BPQ) had substantial loadings on a general factor, resulting in relatively small centre distances. Accordingly, all five of these measures can be considered–more or less distally–to be measures of a global interoceptive sensibility construct. Based on the most centrally located items and scales, we suggest that this global interoceptive sensibility construct can be defined as the tendency to notice bodily changes (across different body systems) in response to an event that might change one’s physiological condition (e.g., consumption of food, a peaceful experience, a change in emotional state; see Section 3.2.1). Given that a range of bodily systems are noted across the five measures, it does not seem to be the case that the core interoceptive sensibility construct is limited to one bodily domain (e.g., cardioception, which has been predominantly focused upon when examining behavioural interoceptive accuracy).

Of the five measures with substantial loadings on the general factor, we found that the items of the MAIA-2 (excluding the Not Distracting and Not Worrying scales) most closely and most precisely tapped the core construct of interoceptive sensibility. That is, while the construct of interoceptive sensibility is multidimensional (as identified by the findings of the present work, and previous work, such as [10]), a score from the six included MAIA-2 scales can be pragmatically considered as a suitable marker of the global interoceptive sensibility construct, particularly for authors facing instrument-selection decisions who are looking to measure this construct in a general sense. Within the MAIA-2, the Emotional Awareness, Self-Regulation, Body Listening, and Attention Regulation subscales are all very centrally located scales, and authors could potentially further select from these scales if necessary. For researchers seeking wider coverage of the interoceptive sensibility construct, the results of the present work suggest that a combination of the MAIA-2, the BAQ, and the PBCS would offer the broadest coverage of the central interoceptive sensibility construct. The addition of the BAQ and PBCS to the MAIA-2 would provide coverage of signals from additional bodily domains (e.g., the gut, which is not covered by the MAIA-2, as in PBCS item #4 “I am quick to sense the hunger contractions of my stomach), and facilitate measurement of the awareness of bodily rhythms and cycles, the ability to detect changes in normal bodily functioning, and the ability to anticipate bodily reactions to various stimuli (i.e., the BAQ items).

Conversely, we found the BRS and BPQ to be more distally located measures of the interoceptive sensibility construct. That is, the aspects of interoceptive sensibility captured by these measures are less central to the core construct that is captured by the MAIA-2, and to a lesser degree by the BAQ and the PBCS. In other words, the BRS and BPQ should be used to examine more specific aspects of interoceptive sensibility, rather than as measures of the global conceptualisation of interoceptive sensibility than aspects tapped by the MAIA-2, the BAQ, and the PBCS. These findings are largely consistent with our hypotheses, and reflect the fact that these measures probe more specific sets of perceptions, emotions and behaviours: the BRS measures the degree to which perceptions of body sensations are used to guide behaviour, and the BPQ examines the awareness of neutral and negative bodily sensations, and less common body sensations such as those associated with illness.

Finally, we found that the IAS and the ISQ were distinct from the core construct, supporting Murphy and colleagues’ [6] theory that the self-reported acuity of interoceptive perception is distinct from self-reported beliefs regarding the tendency to pay attention to interoceptive signals. That is, it is possible for a person to report that they tend to be very aware of internal bodily signals, while also reporting that their perception of these signals is unreliable or inaccurate (e.g., they may feel hungry even after eating a large meal). This distinction, while relatively nascent in terms of being operationalised via self-report measures, has theoretical and clinical utility, particularly when testing theoretical models such as predictive coding models in clinical contexts (e.g., [49]).

The primary limitation of the present work is that the definition of interoceptive sensibility we derived was dependent on the measures we included in the IPV. That is, the inclusion of additional or alternative measures would likely alter the factor structure and, therefore, the location of the centre (i.e., the general factor of all items). While we sought to include some of the most widely cited measures (see [10]), in addition to further measures that would provide broader coverage of the construct, we acknowledge that the measures we included were not exhaustive. Future research could consider whether and where other measures fit within the construct defined here, including recently developed measures such as the Three-domain Interoceptive Sensations Questionnaire [50], which aims to examine awareness of cardiac, respiratory, and gastroesophageal sensations (sample item: “When I make a light physical effort, I notice that my breathing is faster than normal”), and the Interoceptive Attention Scale [35], which aims to examine whether the sensations from the IAS tend to be the object of one’s attention (sample item: “Most of the time my attention is focused on whether my heart is beating fast”). Nevertheless, given that the present study included the most widely cited measures of interoceptive sensibility to date (see [10]), our results make a useful contribution to the current understanding of the interoceptive sensibility construct.

A further limitation of the present work relates to the psychometric properties of the included measures. All of the measures included in the present study had less-than-ideal model fit indices when we examined the fit of the parent models using CFA, which suggests that the measures may deviate from the parent factor structures, at least in the present dataset (e.g., scores on measures that are purportedly unidimensional may, in fact, be better represented by multiple factors). In particular, we were unable to include the Not Worrying and Not Distracting scales of the MAIA-2, as well as three items from the BRS, in the final IPV. Issues with these scales and items are well-documented (e.g., see discussions in [34, 47]) and indeed the MAIA-2 was developed with the intention of improving the psychometric properties of the Not Distracting and Not Worrying subscales [15]. Given the results from the CFA analyses in the present study, we suggest that future researchers should examine the dimensionality of scores on the measures from the present study in their target population. Alternative methods to CFA such as Exploratory Structural Equation Modelling (ESEM) or Bayesian Structural Equation Modelling (BSEM) may be preferential for this work–particularly for the MAIA-2 –because ESEM and BSEM relax the requirements of zero-cross loadings and residual variances ([51, 52], for further discussion, see [53]).

ESEM and BSEM may also be useful for addressing issues associated with negatively worded items (i.e., BAQ Item #10, BRS Items #2, 3, and 4, and the majority of the items from the MAIA-2 Not-Distracting and Not Worrying subscales). While negatively worded items may be useful for minimising acquiescence bias, they also require greater cognitive effort from participants [54]. Accordingly, the inclusion of negatively worded items can commonly lead to the artefactual separation of positive and negatively worded items because of differing response styles to the positive and negative wording, rather than a meaningful distinction between item content [54]. In ESEM and BSEM, it is possible to model correlated uniqueness among items with spurious residual covariances, which allows researchers to better determine whether these items are contributing to ‘noise’ variance that should be eliminated or controlled.

There were additional limitations of the present work which relate to the sample we utilised. While Prolific has been shown to produce higher quality data than other similar online participant recruitment platforms [55], it is important to note that our sample should not be considered representative of the United Kingdom more broadly. Furthermore, we did not collect any data on the physical and mental health status of our sample, or apply any inclusion or exclusion criteria related to this. A wide range of physical and mental health conditions have been associated with differences in interoception (e.g., [1, 56, 57]). It is possible that the presence of unmeasured physical or mental health conditions in our sample could have impacted the present findings, such as by inflating BPQ scores, as many of the BPQ items assess the noticing of uncomfortable body sensations and body sensations associated with illness.

In terms of further future directions, given that the IAS and ISQ were identified as measuring a distinct construct in the present study, and the well-characterised theoretical distinction between attention to interoceptive sensations and accurate detection of interoceptive sensations [6, 7], future research could consider factor commonality and distinguishability among self-report measures of interoceptive accuracy. Such research could include the IAS, the ISQ, and other potential measures of this construct, such as Interoceptive Confusion Questionnaire (which contains items such as “I am very sensitive to changes in my heart-rate” [13]).

To summarise, the present study used IPV–a recently developed illustrative tool based on the comparison of a single factor and correlated factor SEM–to develop a better understanding of scale and item commonality and distinguishability for some of the most frequently cited measures of interoceptive sensibility. Results from the present study support recent postulations that self-report measures of interoception are not measuring a unidimensional construct [6, 10], but nonetheless further suggest that five measures can be grouped together as (more or less distal) measures of a global interoceptive sensibility construct. For authors who are looking to best represent this global interoceptive sensibility construct in a general sense, we recommend use of the MAIA-2, or a combination of the MAIA-2, the BAQ, and the PBCS. More broadly, we recommend IPV as a useful tool to assist decision-making about the utility of particular scales in measuring core constructs.
